# (*E*)-*N*′-(3,4-Dimeth­oxy­benzyl­idene)-4-meth­oxy­benzohydrazide

**DOI:** 10.1107/S1600536812034228

**Published:** 2012-08-25

**Authors:** Muhammad Taha, Humera Naz, Aqilah Abd Rahman, Nor Hadiani Ismail, Yousuf Sammer

**Affiliations:** a Atta-ur-Rahman Research Institute for Natural Products Discovery (RiND), Universiti Tecknologi MARA, Puncak Alam, 42300 Selangor, Malaysia; b Faculty of Pharmacy, Universiti Tecknologi MARA, Puncak Alam, 42300 Selangor, Malaysia; c H.E.J. Research Institute of Chemistry, International Center for Chemical and Biological Sciences, University of Karachi, Karachi 75270, Pakistan

## Abstract

In the title compound, C_17_H_18_N_2_O_4_, the azomethine double bond adopts an *E* conformation with an N—N—C—C torsion angle of −178.3 (3)°. The benzene rings are almost coplaner, with a dihedral angle of 2.98 (14)° between their mean planes. In the crystal, the molecules are linked by N—H⋯O hydrogen bonds, resulting in chains of mol­ecules lying parallel to the *b* axis. The structure is further consolidated by rather weak C—H⋯O hydrogen-bonding inter­actions, resulting in six-membered rings about inversion centers linked into chains arranged parallel to the *b* axis.

## Related literature

For the biological activity of benzohydrazides, see: Bayrak *et al.* (2009 ▶). For the crystal structures of related benzohydrazides, see: Fun *et al.* (2011 ▶); Lu *et al.* (2009 ▶.
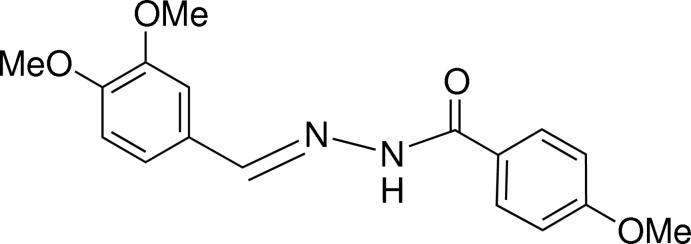


## Experimental

### 

#### Crystal data

C_17_H_18_N_2_O_4_
*M* *_r_* = 314.33
Monoclinic, 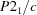

*a* = 14.191 (2) Å
*b* = 5.0109 (8) Å
*c* = 22.535 (4) Å
β = 99.010 (4)°
*V* = 1582.7 (5) Å^3^
*Z* = 4
Mo *K*α radiation
μ = 0.10 mm^−1^
*T* = 273 K
0.23 × 0.20 × 0.04 mm

#### Data collection

Bruker SMART APEX CCD area-detector diffractometer
Absorption correction: multi-scan (*SADABS*; Bruker, 2000 ▶) *T*_min_ = 0.979, *T*_max_ = 0.996
8638 measured reflections
2891 independent reflections
1400 reflections with *I* > 2σ(*I*)
*R*_int_ = 0.080

#### Refinement

*R*[*F* ^2^ > 2σ(*F* ^2^)] = 0.056
*wR*(*F* ^2^) = 0.115
*S* = 0.96
2891 reflections
216 parameters
H atoms treated by a mixture of independent and constrained refinement
Δρ_max_ = 0.18 e Å^−3^
Δρ_min_ = −0.16 e Å^−3^

### 

Data collection: *SMART* (Bruker, 2000 ▶); cell refinement: *SAINT* (Bruker, 2000 ▶); data reduction: *SAINT*; program(s) used to solve structure: *SHELXS97* (Sheldrick, 2008 ▶); program(s) used to refine structure: *SHELXL97* (Sheldrick, 2008 ▶); molecular graphics: *SHELXTL* (Sheldrick, 2008 ▶); software used to prepare material for publication: *SHELXTL*, *PARST* (Nardelli, 1995 ▶) and *PLATON* (Spek, 2009 ▶).

## Supplementary Material

Crystal structure: contains datablock(s) global, I. DOI: 10.1107/S1600536812034228/pv2573sup1.cif

Structure factors: contains datablock(s) I. DOI: 10.1107/S1600536812034228/pv2573Isup2.hkl

Supplementary material file. DOI: 10.1107/S1600536812034228/pv2573Isup3.cml

Additional supplementary materials:  crystallographic information; 3D view; checkCIF report

## Figures and Tables

**Table 1:** Hydrogen-bond geometry (Å, °)

*D*—H⋯*A*	*D*—H	H⋯*A*	*D*⋯*A*	*D*—H⋯*A*
N2—H2*A*⋯O3^i^	0.92 (3)	1.99 (3)	2.890 (4)	166 (2)
C15—H15*A*⋯O4^ii^	0.96	2.54	3.357 (4)	143
C17—H17*A*⋯O2^iii^	0.96	2.55	3.505 (4)	172

Symmetry codes: (i) 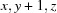; (ii) 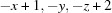; (iii) 
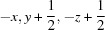
.

**Fig. 1.**
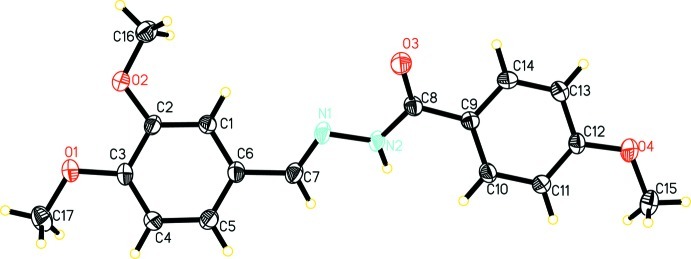
The molecular structure of the title compound with the atom numbering scheme. Displacement ellipsoids are drawn at the 30% probability level. H atoms are presented as small spheres of arbitrary radius.

**Fig. 2.**
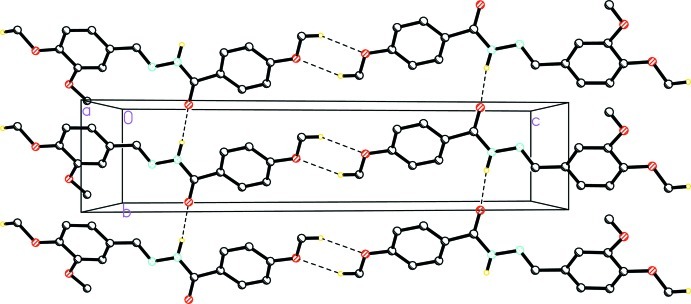
A view of the hydrogen bonding interactions (dotted lines) in the crystal structure of the title compound. H atoms non-participating in hydrogen-bonding were omitted for clarity.

## supplementary crystallographic information

### Comment

Benzohydrazones play an imperative part in research due to their diverse structural features and wide range of biological activities such as antibacterial, antifungal, cytotoxic, anticonvulsant, antiplatelets, anticancer, antinflammatory, and analgesic (Bayrak *et al.*, 2009). The title compound was prepared as a part of our ongoing research on benzohydrazones in order to study their structural features responsible for varoius biological activities.

In the title molecule (Fig. 1), the azomethine double bond (C7═N1, 1.277 (4) Å) adopts an *E* configuration with a torsion angle N2–N1–C6–C7 -178.3 (3)°. The benzene rings (C1–C6 and C9–C14) are almost coplanner with dihedral angle 2.98 (14)° between their mean planes. The bond lengths and angles in the title compound are similar to those reported in structurally realted compounds (Fun *et al.*, 2011; Lu *et al.*, 2009). The crystal structure is stabilized by N2—H2A···O3 intermolecular hydrogen bonds resulting in chains of molecules lying parallel to the *b*–axis (Tab. 1 & Fig. 2). The structure is further consolidated by rather weak intermolecular hydrogen bonding interactions C15—H15A···O4 and C17—H17A···O2, the former resulting in six membered rings about inversion centers and the later forming chains arranged parallel to the *b*-axis.

### Experimental

The title compound was synthesized by refluxing a mixture of 4-methoxybenzohydrazide (0.332 g, 2.0 mmol) and 3,4-dimethoxybenzaldehyde (0.332 g, 2.0 mmol) and a catalytical amount of acetic acid for 3 h in methanol (20 ml). The progress of the reaction was monitored by TLC. After completion of the reaction, the solvent was evaporated by vacuum to afford the title compound (0.496 g, 79% yield). The compound was recrystalize by slow evaporation of a methanol solution to afforded light yellow crystals suitable for single-crystal X-ray diffraction studies. All chemicals were purchased from Sigma-Aldrich Germany.

### Refinement

All C-bound H atoms were positioned geometrically and refined using a riding model, with C—H = 0.93 and 0.98 Å, for aryl and methyl H-atoms, respectively; a rotating group model was applied to the methyl groups. The *U*_iso_(H) were allowed at 1.5*U*_eq_(C methyl) or 1.2*U*_eq_(C non-methyl). The H atom on the nitrogen was located from a difference Fourier map and refined isotropically.

### Crystal data

**Table:**

C_17_H_18_N_2_O_4_	*F*(000) = 664
*M**_r_* = 314.33	*D*_x_ = 1.319 Mg m^−^^3^
Monoclinic, *P*2_1_/*c*	Mo *K*α radiation, λ = 0.71073 Å
Hall symbol: -P 2ybc	Cell parameters from 506 reflections
*a* = 14.191 (2) Å	θ = 2.9–18.7°
*b* = 5.0109 (8) Å	µ = 0.10 mm^−^^1^
*c* = 22.535 (4) Å	*T* = 273 K
β = 99.010 (4)°	Plate, colorles
*V* = 1582.7 (5) Å^3^	0.23 × 0.20 × 0.04 mm
*Z* = 4	

### Data collection

**Table:**

Bruker SMART APEX CCD area-detector diffractometer	2891 independent reflections
Radiation source: fine-focus sealed tube	1400 reflections with *I* > 2σ(*I*)
Graphite monochromator	*R*_int_ = 0.080
ω scan	θ_max_ = 25.5°, θ_min_ = 1.5°
Absorption correction: multi-scan (*SADABS*; Bruker, 2000)	*h* = −17→16
*T*_min_ = 0.979, *T*_max_ = 0.996	*k* = −5→5
8638 measured reflections	*l* = −27→26

### Refinement

**Table:**

Refinement on *F*^2^	Secondary atom site location: difference Fourier map
Least-squares matrix: full	Hydrogen site location: inferred from neighbouring sites
*R*[*F*^2^ > 2σ(*F*^2^)] = 0.056	H atoms treated by a mixture of independent and constrained refinement
*wR*(*F*^2^) = 0.115	*w* = 1/[σ^2^(*F*_o_^2^) + (0.0319*P*)^2^] where *P* = (*F*_o_^2^ + 2*F*_c_^2^)/3
*S* = 0.96	(Δ/σ)_max_ < 0.001
2891 reflections	Δρ_max_ = 0.18 e Å^−^^3^
216 parameters	Δρ_min_ = −0.16 e Å^−^^3^
0 restraints	Extinction correction: *SHELXL97* (Sheldrick, 2008), Fc^*^=kFc[1+0.001xFc^2^λ^3^/sin(2θ)]^-1/4^
Primary atom site location: structure-invariant direct methods	Extinction coefficient: 0.0013 (5)

### Special details

**Table:**

Geometry. All e.s.d.'s (except the e.s.d. in the dihedral angle between two l.s. planes) are estimated using the full covariance matrix. The cell e.s.d.'s are taken into account individually in the estimation of e.s.d.'s in distances, angles and torsion angles; correlations between e.s.d.'s in cell parameters are only used when they are defined by crystal symmetry. An approximate (isotropic) treatment of cell e.s.d.'s is used for estimating e.s.d.'s involving l.s. planes.
Refinement. Refinement of *F*^2^ against ALL reflections. The weighted *R*-factor *wR* and goodness of fit *S* are based on *F*^2^, conventional *R*-factors *R* are based on *F*, with *F* set to zero for negative *F*^2^. The threshold expression of *F*^2^ > σ(*F*^2^) is used only for calculating *R*-factors(gt) *etc*. and is not relevant to the choice of reflections for refinement. *R*-factors based on *F*^2^ are statistically about twice as large as those based on *F*, and *R*- factors based on ALL data will be even larger.

### Fractional atomic coordinates and isotropic or equivalent isotropic displacement parameters (Å^2^)

**Table:**

	*x*	*y*	*z*	*U*_iso_*/*U*_eq_	
O1	0.08439 (16)	0.0973 (5)	0.30744 (8)	0.0533 (7)	
O2	0.02249 (17)	−0.2411 (5)	0.37757 (9)	0.0517 (7)	
O3	0.27009 (17)	−0.4724 (4)	0.67207 (9)	0.0559 (7)	
O4	0.47170 (17)	−0.0216 (5)	0.92189 (8)	0.0597 (7)	
N1	0.23561 (18)	−0.0850 (5)	0.58675 (10)	0.0423 (8)	
N2	0.2763 (2)	−0.0366 (6)	0.64635 (11)	0.0429 (8)	
C1	0.1311 (2)	−0.0799 (6)	0.46462 (12)	0.0383 (8)	
H1B	0.1098	−0.2050	0.4900	0.046*	
C2	0.0930 (2)	−0.0774 (6)	0.40460 (13)	0.0376 (8)	
C3	0.1267 (2)	0.1102 (7)	0.36613 (12)	0.0390 (9)	
C4	0.1959 (2)	0.2892 (6)	0.38871 (13)	0.0426 (9)	
H4A	0.2185	0.4120	0.3634	0.051*	
C5	0.2323 (2)	0.2866 (6)	0.44979 (13)	0.0447 (9)	
H5A	0.2784	0.4110	0.4651	0.054*	
C6	0.2013 (2)	0.1033 (7)	0.48788 (12)	0.0392 (9)	
C7	0.2418 (2)	0.1119 (7)	0.55156 (13)	0.0435 (9)	
H7A	0.2732	0.2658	0.5670	0.052*	
C8	0.2912 (2)	−0.2397 (7)	0.68566 (13)	0.0380 (8)	
C9	0.3389 (2)	−0.1642 (6)	0.74719 (12)	0.0330 (8)	
C10	0.4038 (2)	0.0405 (6)	0.75824 (12)	0.0399 (9)	
H10A	0.4164	0.1460	0.7265	0.048*	
C11	0.4510 (2)	0.0934 (6)	0.81608 (12)	0.0401 (9)	
H11A	0.4957	0.2301	0.8226	0.048*	
C12	0.4308 (2)	−0.0582 (7)	0.86330 (13)	0.0395 (8)	
C13	0.3653 (2)	−0.2629 (7)	0.85314 (13)	0.0468 (9)	
H13A	0.3510	−0.3640	0.8852	0.056*	
C14	0.3212 (2)	−0.3176 (6)	0.79592 (13)	0.0444 (9)	
H14A	0.2787	−0.4597	0.7894	0.053*	
C15	0.5404 (3)	0.1858 (7)	0.93508 (14)	0.0697 (12)	
H15A	0.5626	0.1906	0.9776	0.105*	
H15B	0.5116	0.3540	0.9224	0.105*	
H15C	0.5932	0.1527	0.9142	0.105*	
C16	−0.0236 (2)	−0.4110 (7)	0.41506 (13)	0.0567 (10)	
H16A	−0.0743	−0.5066	0.3909	0.085*	
H16B	−0.0494	−0.3050	0.4442	0.085*	
H16C	0.0218	−0.5356	0.4353	0.085*	
C17	0.1047 (3)	0.3085 (7)	0.26842 (13)	0.0626 (12)	
H17A	0.0644	0.2907	0.2302	0.094*	
H17B	0.1703	0.2987	0.2630	0.094*	
H17C	0.0929	0.4774	0.2860	0.094*	
H2A	0.2850 (18)	0.139 (5)	0.6573 (11)	0.030 (9)*	

### Atomic displacement parameters (Å^2^)

**Table:**

	*U*^11^	*U*^22^	*U*^33^	*U*^12^	*U*^13^	*U*^23^
O1	0.0620 (18)	0.0641 (18)	0.0305 (13)	−0.0093 (14)	−0.0034 (11)	0.0056 (12)
O2	0.0634 (18)	0.0525 (16)	0.0359 (13)	−0.0183 (14)	−0.0021 (12)	0.0002 (12)
O3	0.089 (2)	0.0261 (15)	0.0470 (14)	−0.0012 (13)	−0.0073 (12)	−0.0045 (12)
O4	0.0687 (19)	0.0761 (19)	0.0306 (13)	−0.0173 (15)	−0.0034 (11)	0.0017 (12)
N1	0.057 (2)	0.0356 (19)	0.0302 (15)	0.0044 (15)	−0.0065 (13)	−0.0070 (13)
N2	0.071 (2)	0.0230 (18)	0.0297 (15)	−0.0009 (16)	−0.0083 (13)	−0.0061 (14)
C1	0.049 (2)	0.031 (2)	0.0339 (18)	−0.0020 (18)	0.0027 (15)	0.0015 (15)
C2	0.044 (2)	0.034 (2)	0.0320 (18)	−0.0010 (18)	−0.0022 (15)	−0.0040 (16)
C3	0.042 (2)	0.045 (2)	0.0280 (18)	0.0055 (19)	0.0007 (15)	−0.0007 (17)
C4	0.045 (2)	0.045 (2)	0.0366 (19)	−0.0044 (18)	0.0030 (16)	0.0067 (16)
C5	0.049 (2)	0.040 (2)	0.042 (2)	−0.0053 (18)	−0.0050 (17)	−0.0007 (17)
C6	0.046 (2)	0.037 (2)	0.0331 (18)	0.0037 (18)	−0.0003 (16)	0.0000 (16)
C7	0.054 (3)	0.035 (2)	0.038 (2)	−0.0009 (19)	−0.0042 (17)	−0.0055 (17)
C8	0.050 (2)	0.027 (2)	0.0359 (19)	0.0054 (18)	0.0037 (16)	0.0010 (17)
C9	0.041 (2)	0.024 (2)	0.0337 (18)	0.0031 (16)	0.0054 (15)	−0.0005 (15)
C10	0.050 (2)	0.040 (2)	0.0301 (18)	−0.0014 (19)	0.0058 (15)	0.0014 (16)
C11	0.044 (2)	0.040 (2)	0.0349 (19)	−0.0064 (18)	0.0021 (15)	0.0005 (16)
C12	0.039 (2)	0.049 (2)	0.0289 (18)	0.0020 (19)	0.0016 (15)	−0.0004 (17)
C13	0.055 (3)	0.052 (2)	0.0323 (19)	−0.005 (2)	0.0052 (17)	0.0064 (17)
C14	0.055 (3)	0.033 (2)	0.045 (2)	−0.0077 (17)	0.0053 (17)	−0.0008 (17)
C15	0.077 (3)	0.081 (3)	0.043 (2)	−0.015 (3)	−0.015 (2)	−0.004 (2)
C16	0.051 (3)	0.065 (3)	0.054 (2)	−0.013 (2)	0.0067 (18)	−0.005 (2)
C17	0.073 (3)	0.069 (3)	0.045 (2)	0.008 (2)	0.003 (2)	0.018 (2)

### Geometric parameters (Å, º)

**Table:**

O1—C3	1.366 (3)	C7—H7A	0.9300
O1—C17	1.434 (3)	C8—C9	1.493 (4)
O2—C2	1.362 (3)	C9—C10	1.374 (4)
O2—C16	1.428 (3)	C9—C14	1.395 (4)
O3—C8	1.231 (3)	C10—C11	1.394 (4)
O4—C12	1.369 (3)	C10—H10A	0.9300
O4—C15	1.424 (4)	C11—C12	1.373 (4)
N1—C7	1.277 (4)	C11—H11A	0.9300
N1—N2	1.398 (3)	C12—C13	1.379 (4)
N2—C8	1.344 (4)	C13—C14	1.370 (4)
N2—H2A	0.92 (3)	C13—H13A	0.9300
C1—C2	1.376 (4)	C14—H14A	0.9300
C1—C6	1.395 (4)	C15—H15A	0.9600
C1—H1B	0.9300	C15—H15B	0.9600
C2—C3	1.412 (4)	C15—H15C	0.9600
C3—C4	1.367 (4)	C16—H16A	0.9600
C4—C5	1.393 (4)	C16—H16B	0.9600
C4—H4A	0.9300	C16—H16C	0.9600
C5—C6	1.375 (4)	C17—H17A	0.9600
C5—H5A	0.9300	C17—H17B	0.9600
C6—C7	1.460 (4)	C17—H17C	0.9600
			
C3—O1—C17	117.3 (3)	C9—C10—C11	121.4 (3)
C2—O2—C16	117.9 (2)	C9—C10—H10A	119.3
C12—O4—C15	118.2 (3)	C11—C10—H10A	119.3
C7—N1—N2	113.9 (3)	C12—C11—C10	119.4 (3)
C8—N2—N1	120.0 (3)	C12—C11—H11A	120.3
C8—N2—H2A	123.2 (16)	C10—C11—H11A	120.3
N1—N2—H2A	116.4 (16)	O4—C12—C11	124.4 (3)
C2—C1—C6	120.7 (3)	O4—C12—C13	115.6 (3)
C2—C1—H1B	119.6	C11—C12—C13	120.0 (3)
C6—C1—H1B	119.6	C14—C13—C12	120.1 (3)
O2—C2—C1	125.6 (3)	C14—C13—H13A	119.9
O2—C2—C3	115.0 (3)	C12—C13—H13A	119.9
C1—C2—C3	119.4 (3)	C13—C14—C9	121.2 (3)
O1—C3—C4	124.9 (3)	C13—C14—H14A	119.4
O1—C3—C2	115.0 (3)	C9—C14—H14A	119.4
C4—C3—C2	120.1 (3)	O4—C15—H15A	109.5
C3—C4—C5	119.7 (3)	O4—C15—H15B	109.5
C3—C4—H4A	120.2	H15A—C15—H15B	109.5
C5—C4—H4A	120.2	O4—C15—H15C	109.5
C6—C5—C4	121.2 (3)	H15A—C15—H15C	109.5
C6—C5—H5A	119.4	H15B—C15—H15C	109.5
C4—C5—H5A	119.4	O2—C16—H16A	109.5
C5—C6—C1	118.9 (3)	O2—C16—H16B	109.5
C5—C6—C7	118.6 (3)	H16A—C16—H16B	109.5
C1—C6—C7	122.4 (3)	O2—C16—H16C	109.5
N1—C7—C6	122.2 (3)	H16A—C16—H16C	109.5
N1—C7—H7A	118.9	H16B—C16—H16C	109.5
C6—C7—H7A	118.9	O1—C17—H17A	109.5
O3—C8—N2	123.1 (3)	O1—C17—H17B	109.5
O3—C8—C9	121.9 (3)	H17A—C17—H17B	109.5
N2—C8—C9	114.9 (3)	O1—C17—H17C	109.5
C10—C9—C14	117.8 (3)	H17A—C17—H17C	109.5
C10—C9—C8	123.4 (3)	H17B—C17—H17C	109.5
C14—C9—C8	118.7 (3)		
			
C7—N1—N2—C8	−167.3 (3)	C1—C6—C7—N1	19.1 (5)
C16—O2—C2—C1	7.2 (5)	N1—N2—C8—O3	−0.4 (5)
C16—O2—C2—C3	−171.8 (3)	N1—N2—C8—C9	177.5 (3)
C6—C1—C2—O2	−178.0 (3)	O3—C8—C9—C10	146.6 (3)
C6—C1—C2—C3	1.1 (5)	N2—C8—C9—C10	−31.3 (5)
C17—O1—C3—C4	−8.7 (5)	O3—C8—C9—C14	−29.8 (5)
C17—O1—C3—C2	170.4 (3)	N2—C8—C9—C14	152.3 (3)
O2—C2—C3—O1	−0.7 (4)	C14—C9—C10—C11	0.4 (5)
C1—C2—C3—O1	−179.9 (3)	C8—C9—C10—C11	−176.0 (3)
O2—C2—C3—C4	178.4 (3)	C9—C10—C11—C12	−1.5 (5)
C1—C2—C3—C4	−0.7 (5)	C15—O4—C12—C11	−0.6 (5)
O1—C3—C4—C5	178.6 (3)	C15—O4—C12—C13	179.6 (3)
C2—C3—C4—C5	−0.4 (5)	C10—C11—C12—O4	−179.0 (3)
C3—C4—C5—C6	1.3 (5)	C10—C11—C12—C13	0.8 (5)
C4—C5—C6—C1	−1.0 (5)	O4—C12—C13—C14	−179.3 (3)
C4—C5—C6—C7	−179.6 (3)	C11—C12—C13—C14	0.9 (5)
C2—C1—C6—C5	−0.2 (5)	C12—C13—C14—C9	−2.0 (5)
C2—C1—C6—C7	178.4 (3)	C10—C9—C14—C13	1.4 (5)
N2—N1—C7—C6	−178.3 (3)	C8—C9—C14—C13	177.9 (3)
C5—C6—C7—N1	−162.4 (3)		

### Hydrogen-bond geometry (Å, º)

**Table:**

*D*—H···*A*	*D*—H	H···*A*	*D*···*A*	*D*—H···*A*
N2—H2*A*···O3^i^	0.92 (3)	1.99 (3)	2.890 (4)	166 (2)
C15—H15*A*···O4^ii^	0.96	2.54	3.357 (4)	143
C17—H17*A*···O2^iii^	0.96	2.55	3.505 (4)	172

Symmetry codes: (i) *x*, *y*+1, *z*; (ii) −*x*+1, −*y*, −*z*+2; (iii) −*x*, *y*+1/2, −*z*+1/2.

## References

[bb1] Bayrak, H., Demirbas, N. & Karaoglu, S. A. (2009). *Eur. J. Med. Chem.* **44**, 4362–4366. 10.1016/j.ejmech.2009.05.022 19647352

[bb2] Bruker (2000). *SADABS*, *SMART* and *SAINT* Bruker AXS Inc., Madison, Wisconsin, USA.

[bb3] Fun, H.-K., Promdet, P., Chantrapromma, S., Horkaew, J. & Karalai, C. (2011). *Acta Cryst.* E**67**, o3370–o3371. 10.1107/S1600536811048240 PMC3239014 22199863

[bb4] Lu, J.-F., Min, S.-T., Ge, H.-G. & Ji, X.-H. (2009). *Acta Cryst.* E**65**, o2301. 10.1107/S1600536809034242 PMC2970025 21577690

[bb5] Nardelli, M. (1995). *J. Appl. Cryst.* **28**, 659.

[bb6] Sheldrick, G. M. (2008). *Acta Cryst.* A**64**, 112–122. 10.1107/S0108767307043930 18156677

[bb7] Spek, A. L. (2009). *Acta Cryst.* D**65**, 148–155. 10.1107/S090744490804362X PMC2631630 19171970

